# A Cheek Nodule in a Child: Be Aware of Idiopathic Facial Aseptic Granuloma and Its Differential Diagnosis

**DOI:** 10.3390/ijerph16142471

**Published:** 2019-07-11

**Authors:** Francesco Miconi, Nicola Principi, Lorenzo Cassiani, Federica Celi, Roberta Crispoldi, Ada Russo, Susanna Esposito, Manuela Papini

**Affiliations:** 1 Pediatric Section, Department of Surgical and Biomedical Sciences, Università degli Studi di Perugia, 06132 Perugia, Italy; 2 Pediatric Unit, A.O. Santa Maria Terni, 05100 Terni, Italy; 3 Università degli Studi di Milano, 20122 Milan, Italy; 4 Dermatologic Section, School of Medicine, Università degli Studi di Siena, 53100 Siena, Italy; 5 Dermatologic Section, Department of Surgical and Biomedical Sciences, Università degli Studi di Perugia, 05100 Terni, Italy

**Keywords:** idiopathic facial aseptic granuloma, skin disease, pediatric dermatology

## Abstract

*Background*: Idiopathic facial aseptic granuloma (IFAG) is a rare skin disease that typically presents in children with one or more nontender, erythematous to violaceous nodules located on the cheeks or eyelids. Lesions are not accompanied by other skin abnormalities. IFAG remains a diagnostic challenge in pediatric dermatology, because several diseases may present with similar signs. *Case presentation*: A three-year-old girl with a previous negative clinical history was referred to our hospital for the evaluation of some asymptomatic nodules on the convexity of the left cheek. The nodules had appeared two months before, and had gradually increased in size. Her mother denied any association with trauma or insect bites. The nodules had a hard-elastic consistency, were moderately firm, and were not fluctuant. No associated lymphadenopathy was observed. The girl was afebrile and in good general condition. A histologic evaluation of a biopsy specimen revealed an inflammatory, granulomatous-diffuse infiltrate in the superficial and deep dermis consisting of giant cells, histiocytes, lymphocytes, neutrophils, eosinophils, and plasma cells. The Ziehl–Neelsen stains, Gram-stains, and cultures were negative. Suspecting an IFAG, treatment with topical fusidic acid and oral clarithromycin for 14 days was started. After two months, the lesion resolved and did not recur. *Conclusion*: This case shows how to differentiate IFAG from other dermatologic diseases associated with a negative evolution. Treatment with oral clarithromycin was effective in our patient. However, more scientific evidence is needed to evaluate the most suitable antibiotic therapy. Further studies are also needed to establish whether antibiotics actually impact IFAG prognosis.

## 1. Introduction

Idiopathic facial aseptic granuloma (IFAG) is a rare skin disease that typically presents in children with one or more nontender, erythematous to violaceous nodules located on the cheeks or eyelids [1]. The literature tends to place this condition within the spectrum of childhood rosaceas. Lesions are not accompanied by other skin abnormalities, such as blackheads, telangiectasia, or keratosis pilaris, and are painless. Moreover, they have a soft or elastic consistency with a size of 1 to 3 cm, and tend to resolve spontaneously without scarring after a benign course of a few months to a year [1]. Ultrasound examination usually shows a well-defined hypoechoic solid lesion without calcium deposits, although in some cases, the presence of lobulated margins and an irregular posterior wall with acoustic enhancement has been described [2].

Histologically, IFAG is characterized by a granulomatous tissue reaction in the mid and deep dermis, with histiocytes, lymphocytes, neutrophils, and giant cells, resembling that which is usually found in cases of foreign body penetration or mycobacterial infection [3]. However, none of these potential aetiologic factors has ever been identified by the direct examination of biopsy samples with special and histochemical stains and cultures. Unsuccessful attempts were also made to identify a possible association between IFAG and infectious agents other than *Mycobacterium tuberculosis*.

Although IFAG pathogenesis is presently unknown, two hypotheses have been proposed. It has been suggested that the disease could be a reaction to an embryologic remnant, or against an epidermoid cyst. However, this hypothesis is not supported by histologic evidence. On the contrary, a more attractive theory is the belief that IFAG represents a peculiar variant of childhood rosacea [4]. This is because patients with IFAG are at increased risk of rosacea development, especially ocular rosacea. Moreover, a relevant number of children with IFAG have at least some of the clinical findings, mainly flushing, papules, and pustoles [5], which are considered essential criteria for the diagnosis of rosacea in children [6]. Finally, as in rosacea, some cases of IFAG rapidly respond to local and systemic antibiotic therapy [7].

However, IFAG remains a diagnostic challenge in pediatric dermatology. The clinical course is chronic but benign, and cases have been reported to resolve spontaneously in less than a year. Even though no well-defined treatment has emerged, a conservative approach that avoids aggressive therapies is preferred. The challenge is to diagnose this entity correctly, ideally based on clinical acumen, in order to avoid surgical intervention with facial sutures and the resultant scarring, and unnecessary treatment interventions.

Several diseases may present with similar signs. Differentiation seems mandatory, as some of these diseases can have negative evolution when not diagnosed and treated promptly.

This paper describes a case of IFAG and discusses which diseases can be confused with this benign condition. Moreover, the best therapeutic approach to IFAG is highlighted.

## 2. Case Report

A three-year-old girl with a previous negative clinical history was referred to our hospital for the evaluation of some asymptomatic nodules on the convexity of the left cheek. The nodules had appeared two months before, and had gradually increased in size. Her mother denied any association with trauma or insect bites. Initially considered skin abscesses, they had been treated at home by the primary-care pediatrician with systemic amoxicillin-clavulanate by mouth (50 mg/kg/die as amoxicillin for 10 days) without benefit.

**Figure 1:**
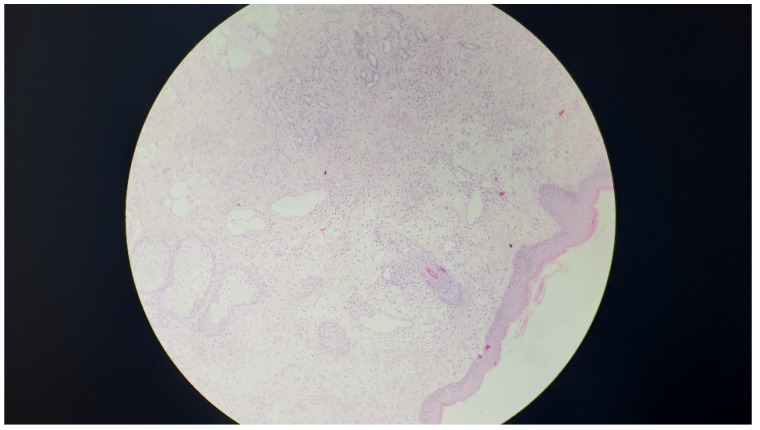
Biopsy of the cheek nodule. A histologic examination revealed a moderately well-defined granuloma in the superficial and deep dermis mainly composed of giant cells. Some eosinophils, histiocytes, and lymphocytes can be observed in the lesion.

At admission, the examination of the cheek revealed two nodules one centimeter in diameter, and another two centimeters in diameter with overlying erythematous skin and negative thermotouch. The nodules had a hard-elastic consistency, were moderately firm, and were not fluctuant. No associated lymphadenopathy was observed. The girl was afebrile and in a good general condition. An ultrasound examination performed using a 12 Hz linear probe showed that the lesions were homogenous, hypoechoic, and solid. A histologic evaluation of a biopsy specimen of the largest nodule revealed an inflammatory, granulomatous-diffuse infiltrate in the superficial and deep dermis consisting of giant cells, histiocytes, lymphocytes, neutrophils, eosinophils, and plasma cells (Figure 1). The Ziehl–Neelsen and Gram stains were both negative. The cultures for the anaerobic and aerobic bacteria, fungi, and acid-fast bacilli were negative, as they were the antibody tests for *Bartonella henselae* and *Afipia felis*.

**Figure 2:**
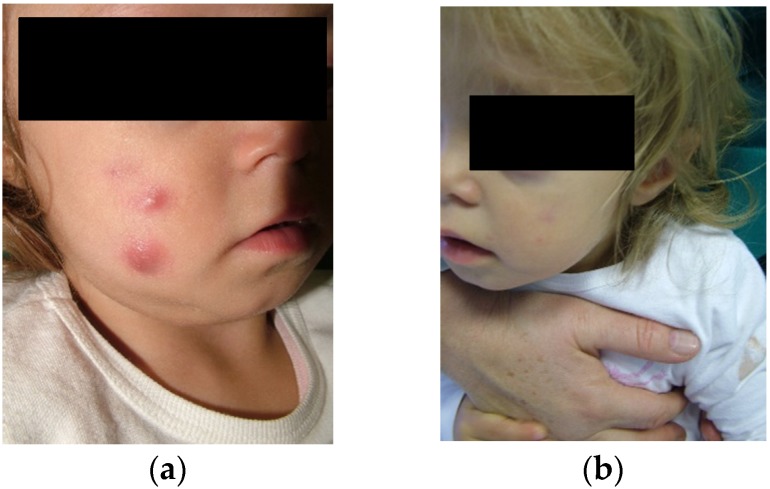
Skin nodules (**a**) before and (**b**) at the end of clarithromycin treatment.

Suspecting an IFAG, treatment with topical fusidic acid and oral clarithromycin for 14 days was started, and a monthly follow-up was scheduled. Figure 2 shows skin nodules before and at the end of treatment. After two months from the first evaluation, the lesion resolved and did not recur after one additional month.

The management of this patient was approved by the Ethics Committee of Umbria Region (PED-2018-12), and both parents provided written informed consent for the evaluation of the child. The Ethics Committee of Umbria Region approved the publication of this case, and both parents provided written informed consent for the publication of this manuscript, including photos.

## 3. Discussion

The clinical manifestations, ultrasound findings, and histology of the case reported here are consistent with the first descriptions of IFAG cases, and confirm the diagnosis and the peculiarities of this disease [8], which is characterized by one or few painless red to violet nodules that grow slowly on the cheek and/or on the eyelids. However, diagnosis is difficult, as acquired nodules on the face can be clinical manifestations of several diseases.

Table 1 summarizes the main differential diagnoses of IFAG, such as chalazion, nodular infantile acne, localized infectious pyodermas, pyogenic granulomas, xanthogranulomas, vascular malformations, dermoid or epidermoid cysts, and benign tumors such as pilomatricomas [9,10,11,12]. In some patients, the differentiation of these diseases from IFAG can be made only based on the medical history and clinical manifestation characteristics. However, in several other cases, laboratory tests, ultrasonography, and histology are needed. Chalazion should be considered when IFAG is on the eye. It is a cyst formed as a result of a blocked oil gland, and is red and painless [9]. When the Meibomian glands are blocked, the oedema is ordinarily contained in the conjunctival portion of the lid. In contrast, chalazion because of the blockage of the Zeis glands is usually located along the lid margin [9]. Nodular infantile acne is characterized by the presence of several superficial inflammatory papules and blackheads, and causes significant problems of differentiation from IFAG only when the nodules are very few and on one cheek [10]. Pyogenic granuloma is a benign vascular tumor that is composed of capillaries and venules, and appears as small or large, reddish exophytic vascular nodules that can grow rapidly and are smooth or lobulated [11]. Pyogenic granuloma often involves the gums, nasal septum, and skin [11]. Epidermoid, dermoid, and teratoid cysts of the face can be confused with IFAG, but ultrasonography and biopsy can solve the diagnostic problem. The same is true for juvenile xanthogranuloma. This is a rare and benign condition that is included in the group of non-Langerhans histiocytosis, and is characterized by the presence of papules or yellow and/or erythematous nodules than can be asymptomatic, multiple, or solitary [12]. Bacterial, fungal, and parasite infections are usually easily distinguished from IFAG, because in this disease, no sign of inflammation and no modification of laboratory tests can be demonstrated.

When the diagnosis of IFAG is established, two problems remain. The first is whether the patient must be referred to an ophthalmologist because of the potential relationship between IFAG and the subsequent development of ocular rosacea. This is suggested by some authors, particularly in the case of nodules on the eyelids or an association with chalazion. Eye involvement in the case of rosacea includes blepharitis with meibomian gland inflammation and relapsing chalazions, ocular redness, photophobia, episcleritis, or keratoconjunctivitis, and, although rarely, corneal ulcers can lead to severe ocular infection and visual impairment [6]. The second is whether an antibiotic therapy should be prescribed. In some patients, an apparent faster reduction of nodules has been described. In other cases, treatment was apparently effective, although, as in the patient described here, only after a second antibiotic course [3,13,14].

The choice treatment of IFAG is conservative, and surgery should be avoided, given the benign and self-healing character of the lesion. Antibiotic treatment is still discussed, as some authors consider it ineffective. Treatment with oral doxycycline for rosacea in the age range ≥8 years has been proposed. In younger children, oral macrolides such as clarithromycin or azithromycin and metronidazole in cream have been attempted [15]. The oral clarithromycin had good results in our case with an excellent tolerability profile, and could be considered in the treatment of this pathology.

## 4. Conclusions

This case shows how to differentiate IFAG from other dermatologic diseases associated with negative evolution. Treatment with oral clarithromycin was effective in our patient. However, more scientific evidence is needed to evaluate the most suitable antibiotic therapy. Further studies are also needed to establish whether antibiotics actually impact IFAG prognosis.

## Figures and Tables

**Table 1:** Differential diagnosis of idiopathic facial aseptic granuloma (IFAG).

Skin Lesion	Site	Clinic	Aetiology
IFAG	Nodules located on the cheeks or eyelids.	One or more nontender, erythematous to violaceous nodules.	Still unclear.
Chalazion	Conjunctival portion of the lid.	Red and painless.	Cyst because of a blocked oil gland.
Nodular infantile acne	Face and trunk.	Several superficial inflammatory papules and blackheads.	Chronic-inflammatory disorder of the hair follicle and sebaceous glands.
Pyodermas	Usually lower limbs or trunk.	Recurrent appearance of large skin ulcers.	Unknown, probably depends on an abnormal immuno-mediated response.
Pyogenic granulomas	Gums, nasal septum, or other sites on the skin.	Reddish exophytic vascular nodules that can grow rapidly.	Benign vascular tumor that is composed of capillaries and venules.
Xantogranulomas	Head, neck, or trunk.	One or multiple brown-yellow nodules.	Can be associated with glaucoma, uveitis or iritis, and Von Recklinghausen’s disease.
Vascular malformations	Anywhere along the blood vessels.	Variable, the flat angioma presents itself as a congenital macula, pale pink to vinous red, and variable extension and shape.	Abnormal development of capillaries, arteries, veins, and lymphatic vessels.
Pilomatricomas	Face and neck, in particular the preauricular area, cheek, forehead, upper eyelid, and eyebrow.	Solitary neoformed nodules asymptomatous of stony hardness of irregular shape, angled and faceted.	Keratin originating from the hair bulb derivated from the Trichocytes.
Dermoid and epidermoid cysts	Head, neck, or face.	Mass that normally becomes visible at birth or in early childhood as a small painless lump.	Congenital defect that is created during the development of the embryo for a defective growth of the skin layers.
